# Urban and peri-urban family-based pig-keeping in Cambodia: Characteristics, management and perceived benefits and constraints

**DOI:** 10.1371/journal.pone.0182247

**Published:** 2017-08-16

**Authors:** Gunilla Ström, Agnes Andersson Djurfeldt, Sofia Boqvist, Ann Albihn, Seng Sokerya, Sorn San, Holl Davun, Ulf Magnusson

**Affiliations:** 1 Department of Clinical Sciences, Swedish University of Agricultural Sciences, Uppsala, Sweden; 2 Department of Human Geography, Lund University, Lund, Sweden; 3 Department of Biomedical Sciences and Veterinary Public Health, Swedish University of Agricultural Sciences, Uppsala, Sweden; 4 National Veterinary Institute, Uppsala, Sweden; 5 Centre for Livestock and Agriculture Development, Phnom Penh, Cambodia; 6 National Veterinary Research Institute, Phnom Penh, Cambodia; Public Library of Science, FRANCE

## Abstract

Keeping pigs in urban and peri-urban areas may not only provide many benefits for the urban households, but may also be challenging and a potential health hazard. The aim of this cross-sectional study was to describe household characteristics and to evaluate perceived benefits and constraints among pig-keepers in the urban and peri-urban areas of Phnom Penh, Cambodia. The study included 204 households and a structured questionnaire was used to interview the household member responsible for taking care of the pigs. Descriptive analyses showed that most households kept between 5 and 15 pigs and that all households kept their pigs in confinement. About 97% of the households owned the pigs themselves and the pigs were generally managed by female household members (43%). Pigs were mainly kept for commercial purposes and more than 60% of the households stated that income from pig-keeping was the main or one of the main sources of revenue for the household. More than 82% reported that they had experienced disease outbreaks among their pigs during the past three years and disease outbreaks were more commonly reported in households with lower socio-economic position (P = 0.025). Disease outbreaks were considered one of the main constraints, along with expensive feed and low payment prices for the slaughter pigs, but few households considered sanitary or other public health issues problematic. Thus, pig-keeping makes an important contribution to the livelihoods of urban and peri-urban households, but many households face external constraints on their production, such as diseases and low revenues, which may have a negative impact on their livelihoods.

## Introduction

Keeping livestock in urban and peri-urban areas is a common practice in many low-income countries. The driving force appears to be increased migration from rural to urban areas, along with increased demand for animal-based foods [1]. Simultaneously, there is an urbanisation of poverty [2,3], and the occurrence of food insecurity and malnutrition is a prevailing problem among the urban poor [4,5].

Keeping livestock in urban and peri-urban areas can be an important survival strategy for the urban poor [6,7]. Keeping livestock may offer an opportunity to improve the quality of life through increased cash income from sales, and through improved nutrition [8,9]. Improved nutrition is attained as these households are likely to consume a diverse and nutritious diet, including animal-based foods [7,10,11]. As diets change, as a consequence of urbanisation and rising income [1], urban livestock production may also, to some extent, meet the increased urban demand for animal-based foods, and may complement rural and foreign sources of food supply to cities. It has been estimated that urban and peri-urban livestock production actually represents more than one-third of the world’s total meat production [12].

Even though livestock-keeping may contribute to the livelihoods of the urban poor, keeping livestock in urban areas may be controversial. For instance, there are rising concerns about the public health risks associated with keeping livestock in densely populated areas. These concerns include transmission of zoonotic diseases [13,14], and sanitary aspects of smell and pollution, caused by inadequate handling of livestock manure [15,16]. Inadequate handling of manure may also attract insects and rodents, which may further increase the risk of disease transmission [17].

Keeping livestock, especially pigs, is a common practice in Southeast Asia [18]. Pigs require low inputs of labour and feed and pig-keeping can easily be combined with domestic work, providing a potential source of empowerment for women. To date, research has been conducted on urban and peri-urban pig-keeping in Africa [19–21], whereas literature regarding the Southeast Asian region is scarce. Given the important role of pig-keeping in Southeast Asia, and the rapid urbanisation in the region [3], we here try to reduce that knowledge gap. In this study, pig-keeping households in the urban and peri-urban areas of Phnom Penh, Cambodia, were surveyed with respect to household characteristics, including gender disparities and livestock management. The objectives were to describe the characteristics of these households and to assess the benefits and constraints pig-keepers perceive in urban areas.

## Materials and methods

### Study area

The study area comprised urban and peri-urban areas of Phnom Penh, south Cambodia, with an estimated population of approximately 1.7 million inhabitants [22]. During the past two decades, Cambodia’s economy has been among the fastest growing in the world, with substantial improvements in human development and poverty reduction. Along with these improvements, the urban population has increased, with an estimated annual growth rate of 2.5% between 2010 and 2014, compared with 1.6% at national level [22].

### Study design and data collection

Data were collected in November and December 2014 and in February 2015. The households were geographically evenly distributed in both data collection periods. Initially, a census of households keeping pigs in Phnom Penh was performed by a team composed of staff from the Centre for Livestock and Agriculture Development (CelAgrid), and students from the Royal University of Agriculture (RUA), Phnom Penh. The procedure used was snowball sampling [23], where the team used different starting points in order to cover the entire city, with the aim to locate all pig-keeping households in Phnom Penh. In total, 267 households keeping pigs as a family farm were found. Family farms were defined as being managed and operated by the family members, which is consistent with the definition by the Food and Agriculture Organization of the United Nations (FAO) [24].

In the main study, all households were visited by the first author and an interpreter, and their inclusion was based on whether the family was at home when visited and on their willingness to participate, which resulted in a total of 204 households. Fourteen households were unwilling to participate, and in 20 households the family was not at home during any of the days the area was visited. Twenty-one households had stopped keeping pigs since the first team visited the household during the census, and six households could not be located again. All households were allocated an identification code and the geographical position of each participating household was recorded using a handheld global positioning device (GPS-Garmin eTrex H).

A structured questionnaire with a combination of dichotomous, multiple choice and open-ended questions was developed. The questionnaire was in English and included questions on demographics, socio-economic characteristics, livestock management and the respondent’s experiences and perceptions about their pig production. The questions regarding perceived benefits and constraints to pig keeping were only posed to half of the participating households (n = 102), due to logistic reasons. This was done during the second collection period (February 2015), and the households were geographically evenly distributed.

A draft of the questionnaire was pre-tested in households close to Phnom Penh, in the adjacent province, and the questionnaire was then refined on basis of the feedback from the pre-testing sessions. The final questionnaire took around 30 minutes to complete and targeted the person taking care of the pigs. Interviews were performed in the native language Khmer and were carried out with assistance of the same interpreter throughout the study. Questionnaires were checked for accuracy immediately after the interviews.

### Ethics statement

All participating households were informed about the purpose of the study and that their participation was voluntary and anonymous. Verbal consent was obtained from each household and documented in the questionnaire. Prior to the study, statements were received from the National Institute of Public Health (NIPH) in Cambodia and the Regional Ethical Review Board in Uppsala, Sweden, that the study procedure was approved according to their respective standards regarding ethics in research involving humans [25].

### Data management

Data from questionnaires were transcribed into the software Epi Info^™^ 7 (CDC, Atlanta, GA) by the first author within a day of the interviews. The data were then exported to Microsoft Office Excel 2010 spreadsheets after the fieldwork was finished.

#### Calculation of socio-economic position

A wealth index was calculated for each household to define its socio-economic position [26,27]. Participants were asked questions about home ownership and construction of dwelling, availability of agricultural land, and ownership of consumer durables and other household belongings. Descriptive analyses were performed for all variables intended for the index, and variables showing low frequencies were excluded as they would get too small weights in the final index. Principal component analysis (PCA) was used to assign weights to the different variables, as described in Vyas and Kumaranayake [28]. IBM SPSS Statistics for Windows, version 22.0 (Armonk, NY: IBM Corp.) was used for the PCA. Dummy binary variables were created for all categorical variables, to capture most of the variance between variables in the index and to avoid clustering of data [29].

From the final set of correlated variables, PCA was used to create uncorrelated components, where the first component explained the largest amount of variation in the data. This component was used as a score representing each variable’s contribution to the household’s socio-economic index. From the factor score, a dependent variable was constructed for each household, with a mean equal to zero and a standard deviation equal to one, which was regarded as the socio-economic score of the household. The socio-economic index was later included in the regression models as a continuous independent variable. The index should be interpreted as a survey-specific wealth indicator, since it only measured the relative socio-economic position within the study population. Due to missing data, one of the households was not included in the index calculations.

#### Adjusting household size

A household was defined as all members of a common decision-making unit that were sharing income and other resources. The size of the household was adjusted to consider the age distribution of the family members, in order to capture the household’s volume of consumption. Calculations were performed according to Andersson Djurfeldt [30], where all adult members (aged 16 to 60 years) were assigned a value of 1, and children (15 years and below) and the elderly (61 years and above) were assigned a value of 0.5 and 0.75, respectively. For each household, a score was subsequently calculated.

#### Determining the size of the pig production

To determine the size of each household’s pig production, information was collected on the current number of pigs in the household, at the time of visit, as well as the number of pigs that had been born in or purchased by the household in the past 12 months. The latter is referred to as ‘inflow’ in the text.

### Statistical analyses

Statistical analyses were conducted in SAS software 9.4 (SAS Institute Inc., Cary, NC). Descriptive statistics were computed for household characteristics and livestock management. Distributions for the variables ‘socio-economic index’ and ‘inflow of pigs’ were tested for normality, using Shapiro-Wilks test, where the latter was not normally distributed. Kruskal-Wallis test and pairwise two-sample Wilcoxon test were used to test differences in ‘inflow of pigs’ in each household with type of pig production system practised in the household, and with gender differences in responsibility for animals. To test whether the socio-economic index differed between male- and female-headed households, an independent two-sample t-test was used. Univariable logistic regression was used to investigate potential associations between socio-economic index and reported disease occurrence among pigs. Furthermore, Chi-square and Fisher’s Exact test were used to investigate gender differences in education level, and to investigate associations between disease occurrence among the pigs, the practice of vaccination of pigs and whether disease was considered a constraint or not. The statistical significance level was defined as a two-tailed p-value ≤0.05 for each model.

## Results

### Household characteristics

The non-adjusted household size ranged from 1 to 9 members, with a median size of 5.0 (5^th^ and 95^th^ percentiles: 3.0 and 7.0). The adjusted household size ranged from 1 to 8.5, with a median of 4.0 (5^th^ and 95^th^ percentiles: 2.5 and 6.7).

Households most commonly consisted of an adult couple (<60 years old) with young or grown-up children (65%). In addition, 17% of the households also had children’s spouses and grandchildren, as well as other relatives, living in the house. Seven percent of the households consisted of an elderly couple (>60 years old) with other family members, such as children, their spouses and grandchildren. If the household consisted of an adult couple, the man was designated by family members as the head of the household. In the nine households that were designated as female-headed, most women were widows (89%).

Only 17% of the heads of the households responded that pig-keeping was their main occupation (Table 1). The most common occupation for the head of the household was employment outside the household, practised by 32%. Other common occupations were rice wine production in combination with pig-keeping (24%), and mixed farming with both livestock and crop production (13%).

**Table 1:** Household characteristics among pig-keepers in urban and peri-urban Phnom Penh (n = 204), (Cambodia 2014–2015).

Variable	Category	n	%
Main occupation of household head*			
	Employment outside household	65	32
	Combined pig and rice wine production	49	24
	Pig-keeping	34	17
	Mixed crop and livestock	26	13
	Fishing	14	7
	Crop production	5	2
	Cattle production	2	1
	Poultry production	2	1
	None	7	3
Completed higher education**			
	Males^a^ (n = 337)	135	40
	Females^b^ (n = 302)	63	21

*Household head defined by the household.

**Higher education defined as upper secondary school and above.

^a-b^ Means within a column with different superscripts differ (P<0.01).

The socio-economic index of households ranged from -3.44 to 3.08. There was no significant difference in index between male- and female-headed households. The education level of the head of the household was not correlated with the index score, but men on average had a higher education level than women (P<0.01) (Table 1). Higher education was classified as upper secondary school and above, which was completed by 40% of the men, compared with 21% of the women.

Almost half of the households (48%) had access to agricultural land, of which 84% owned all or some of that land. The area of land ranged from 0.002 hectares (ha) to 6 ha, and most households (85%) had access to1 ha or less. The land was most commonly used for rice production (74%).

### Livestock management

Around 11% of the households had started their pig production during the past year, whereas 38% had been raising pigs for more than 10 years. In 39% of the households, at least one adult member had some previous experience of keeping pigs before they started their own production, of whom 93% had gained their experience from their parents.

#### Animal species and housing

Of the 204 participating households 33% also kept cattle, 77% kept chickens and 32% kept ducks. One household owned a buffalo. The number of pigs present in the household at the time of the visits ranged from 1 to 200, with a median of 12 (5^th^ and 95^th^ percentiles: 2 and 57). Most households kept between 5 and 15 pigs. The number of pigs born in or purchased by the household during the past 12 months (‘inflow’) ranged from 2 to 360, except for one household where 703 pigs had been born during the past 12 months. The median annual inflow of pigs was 30 (5^th^ and 95^th^ percentiles: 8 and 100), and most households (81%) had an inflow of 50 pigs or less (Table 2).

**Table 2:** Inflow of pigs (frequency groups) in the participating households during the past 12 months (n = 204), (Cambodia 2014–2015).

Frequency group	No. of households	%
1–5	8	4
6–10	16	8
11–20	36	18
21–30	61	30
31–40	29	14
41–50	16	8
51–100	28	14
>100	10	5

The type of pig production system varied between households (Table 3). In ‘fattener production’, practised by 37% of the households, one-month-old piglets were purchased from other farms or households and raised for slaughter. Households that produced and sold piglets, ‘piglet producers’, kept sows that gave birth to about two litters of piglets a year and accounted for 7% of the participating households. One third (34%) applied a ‘farrow-to-finish’ system, where the piglets born were raised for slaughter in the same household. The remaining households applied different combinations of these systems. Piglet-producing households and households with a combination of ‘farrow-to-finish’ and ‘piglet production’ had a higher inflow of pigs than the other production systems (P<0.05), although one piglet-producing household had an annual inflow of 703 pigs, which was much higher than for the other households. However, even when this outlier household was removed from the analysis, the difference between the production systems was still significant (P<0.05).

**Table 3:** Mean annual inflow of pigs in the participating households, based on production system and responsibility for taking care of the pigs (n = 204), (Cambodia 2014–2015).

Variable	Category	No. of households (%)	Mean annual inflow of pigs*	SD**
Production system				
	Fattener production	76 (37)	28^a^	20
	Farrow-to-finish	69 (34)	37^a^	49
	Piglet production	14 (7)	112^b^	174
	Combination farrow-to-finish and piglet production	14 (7)	119^b^	106
	Combination farrow-to-finish and fattener production	31 (15)	36^a^	20
Responsible for taking care of the pigs***				
	Male	59 (29)	39	33
	Female	87 (43)	36	42
	Shared	56 (28)	64	110

*Mean number of pigs born in or purchased by the household in the past 12 months.

**SD = Standard deviation.

***Variables missing from two households (n = 202).

^a b^ Means within a column with different superscripts differ (P<0.05).

No households kept their pigs free roaming, but all pigs were kept inside pens except in two households, where pigs were tied up temporarily due to flooding and construction of new pens. However, young piglets could often move freely between and outside pens. Cattle were most commonly tethered (94%), while chickens and ducks were either kept free roaming in the household (36%), or in a combined system where they were kept in an enclosure during the night (63%). In the majority of households, interspecies interactions were possible.

#### Ownership and responsibility

Ninety-seven percent of the households owned the pigs themselves. The remaining six households reported that they owned some of the pigs or that children who had moved out of the household owned the pigs. In 43% of the households, women were responsible for the daily management of the pigs. Men were responsible in 29% of the households while in 28% the responsibility was shared between household members (Table 3).

### Perceived benefits from pig-keeping

Increased income was reported by all households as one of the most important reasons for keeping pigs, as all households reported that they mainly kept pigs for sale and not for their own consumption. More than 60% reported that the pig production represented one of the main income sources for the household, followed by income from employment outside the household (18%). Only eight households (4%) relied entirely on pig-keeping for income generation.

Other reasons for keeping pigs were that they could act as an economic reserve, and that keeping pigs entailed some degree of freedom, as pig-keeping could be combined with other occupations, such as rice wine production and crop production. The possibility to use residues from other types of agricultural production as feed was mentioned as an important reason for keeping pigs. In fact, residues from rice wine production and residues from nearby breweries were used as pig feed by 45% and 13% of the households, respectively. Some households engaged in crop production also mentioned the benefit of using pig manure as fertiliser, although this comprised only 6% of all households.

### Perceived constraints to pig-keeping

Fifty percent of the households considered diseases to be one of the main constraints to pig-keeping (Table 4). More than 82% of the households reported outbreaks among their pigs during the past three years, and outbreaks were significantly more common in households with a lower socio-economic index (P = 0.025; Odds Ratio (OR) = 1.55; 95% Confidence Interval (CI) = 1.06–2.27). Common diseases, which had been diagnosed by veterinarians, were Foot and Mouth Disease (FMD), Pasteurellosis, Porcine Reproductive and Respiratory Syndrome (PRRS), Aujeszky’s disease and Classical Swine Fever. Most households (95%) vaccinated their pigs against one or more of these diseases, but there was no association between the practice of vaccination and reported disease outbreaks. Households that reported disease outbreaks were more likely to cite diseases as a constraint (P = 0.012; OR = 5.47; CI = 1.45–20.6). Respondents also considered the current low prices for slaughter pigs a major constraint, and a large proportion of households believed import of pigs from neighbouring countries to be the main cause of this problem. High cost of feed was also identified as an important constraint, limiting households from purchasing enough feed of sufficient quality. A few households also mentioned the problem of manure management and that the smell of manure sometimes caused conflicts with neighbours. Fourteen percent could not think of any constraints to pig-keeping.

**Table 4:** Perceived constraints to pig-keeping amongst the households studied (n = 102)* (Cambodia 2014–2015).

Constraint	No. of households	%
Low prices for slaughter pigs	55	54
High cost for feed	54	53
Diseases among the pigs	51	50
Expensive to buy piglets	9	9
Smell of manure	4	4
Low fertility of sows	2	2
Lack of capital	2	2
Dependent on market prices	2	2
No constraints	14	14

*Multiple answers were possible.

## Discussion

This study showed that urban and peri-urban households in Phnom Penh mainly keep pigs to provide extra income for the household. Disease outbreaks among pigs were considered a major constraint, as were high prices of pig feed and low prices for slaughter pigs.

Households surveyed were mainly smallholders, where the majority kept between 5 and 15 pigs. In general, pigs in Cambodia are kept in extensive small-scale systems [31], and several studies in rural areas have reported that most households keep less than 5 pigs [32,33]. However, the households in this study had a median of 12 pigs, which suggests that family farms around Phnom Penh on average keep more pigs than in rural areas of Cambodia. Similar differences between urban and rural areas have been observed in Nigeria, e.g. a study in a peri-urban area of southwestern Nigeria found that 60% of pig-keepers had more than 50 pigs [21], whereas households in various rural areas in Nigeria have been reported to have a mean number of between 2 and 4 pigs [34]. In the Democratic Republic of the Congo, the average herd size was twice as large in peri-urban areas as in rural areas [20]. A possible explanation could be that households closer to urban areas are more market orientated, as a result of better infrastructure and proximity to traders and slaughterhouses.

The suggestion that urban and peri-urban pig-keepers are more market orientated is reinforced by the finding that all households in this study mainly kept pigs for sale, rather than for subsistence. The purpose of keeping pigs also appears to vary between countries and between urban and rural areas. Pigs in rural areas of Cambodia [32] and Lao PDR [35] have been reported to be mostly raised for sale, whereas smallholder pig farms in Vietnam [36] and Nigeria [20] tend to be less market orientated the farther away from town they are located.

Households included in this study were all family farms, with pig production managed and operated by family members. Women were most commonly responsible for taking care of pigs, which is consistent with studies in Cambodia [32] and Lao PDR [37]. In the Democratic Republic of the Congo, however, Kambashi et al. [20] found that only 11% of peri-urban pig farms were under the supervision of women, a division of labour also reported in Cameroon [19]. The latter study found that although men were often responsible for the strenuous and tedious work with the pigs, women were still often involved in other daily operations, such as feeding and cleaning.

Most households in the present study mentioned increased income as the main reason for keeping pigs. Although the majority reported that pigs were the main or one of the main income sources for the household, few relied solely on pig-keeping for income. This is consistent with findings from a study in urban and peri-urban areas of Kampala, Uganda, where none of the households was wholly economically dependent on pigs [38]. Furthermore, Fualefac et al. [19] reported that almost 70% of peri-urban pig-keepers in Cameroon primarily raised pigs for marketing and income generation, whereas 18% mainly wanted to get rid of kitchen and farm waste. The latter reason was also mentioned by some households in our study.

Almost half of the pig-keeping households in Phnom Penh were producing rice wine, from which they obtained residues of fermented rice that were fed to the pigs. A study in three rural provinces of Cambodia found that rice wine residues were fed to pigs by only 3–8.5% of the participating households [33]. Using rice wine residues as pig feed may be more common in urban areas in Cambodia due to better market opportunities for rice wine in those areas. Furthermore, brewery residues were fed to pigs in 13% of the households in our study, whereas this practice was not reported for any rural households [33].

Half of the households in this study considered infectious diseases to be one of the main constraints to their pig production, a perception that has also been reported among smallholder pig-keepers in rural Cambodia [33] and Lao PDR [35]. Households that had experienced disease outbreaks during the past three years were more likely to consider diseases a problem in their production, most likely because they were aware of the consequences. Furthermore, disease outbreaks were more commonly reported by households with lower socio-economic position. A possible explanation is that these households may not have enough capital to invest in preventive measures, or that they are forced to use lower quality feeds that are more often contaminated with pathogenic microorganisms.

Keeping pigs in confinements, thus avoiding extensive contact with other animals, is an important preventive measure for disease transmission [17]. This was practised by all households in this study, whereas in rural households in Cambodia, scavenging systems are more common [32,33]. Similar differences between urban [19] and rural [39] areas in Cameroon, and Uganda [40], have been observed. In this respect, biosecurity might in fact be better on urban and peri-urban pig farms, than on their rural counterparts. On the other hand, the density of animals and humans is much higher in urban and peri-urban areas, which may facilitate disease transmission [41]. Furthermore, we found that poultry and other animals were often kept free roaming and could easily come into contact with pigs and move between households. The practice of keeping other animals free roaming might hence increase the risk of transmission of diseases that can affect different species, even though the pigs are confined. In addition, traders collecting pigs for slaughter, or delivering piglets, might be a significant biosecurity risk, as might the practice of slaughtering animals or handling slaughter waste in the household [17], although these practices were not included in this study.

High prices of feed and low prices per unit live weigh for pigs were also considered major constraints among urban and peri-urban pig-keepers in Phnom Penh, resulting in decreased revenue and competitiveness. Similar perceptions have previously been reported amongst rural pig-keepers in Cambodia [31]. Large imports of live pigs from Thailand and Vietnam have been argued to be a contributing factor to the unstable market prices and the less appealing pig industry in Cambodia [31,42]. The issue of pig imports from neighbouring countries was also mentioned by many households in our study as one of the major causes of unstable market prices.

Interestingly, not a single household mentioned concerns about food safety or disease transmission from pigs to humans. The three most commonly mentioned challenges and constraints to their pig production related directly to lost income or higher production costs. The answers might reflect the limited knowledge about food safety and biosecurity among the pig-keepers studied. In addition, food safety might not be a primary concern regarding people’s perceptions about their livelihoods and is often to some extent neglected when considering food security [43]. It is notable, however, that no problems regarding zoonotic disease transmission were mentioned by the pig-keepers, although this is a concern often raised by various stakeholders internationally [44].

Although the aim of the census was to locate all pig-keeping households in Phnom Penh, it cannot be guaranteed that all households have been included. However, as the team that conducted the census covered the majority of the city province and the included households were fairly evenly distributed geographically, we do consider the results to give a representative sample of the pig-keepers in the region.

## Conclusions

Pig-keeping in urban and peri-urban areas of Phnom Penh is mainly pursued for income generation and the majority of households studied considered income from pigs to be one of the most important sources of revenue for the household. Outbreaks of diseases and low revenues were the main perceived constraints, whereas few households considered sanitary or other public health issues to be problematic.

## Supporting information

S1 Questionnaire Urban and peri-urban livestock-keeping in Cambodia. (DOCX)

S1 Table Self-reported household belongings and score of the principal component from the PCA used for the socio-economic index calculations. (DOCX)

S1 File Data from questionnaires relevant for this paper. (XLSX)

## References

[1] Popkin BM. Urbanization, lifestyle changes and the nutrition transition. World Dev. 1999; 27: 1905–1916. doi: 10.1016/S0305-750X(99)00094-7

[2] Ravallion M, Chen S, Sangraula P. New evidence on the urbanization of global poverty. Popul Dev Rev. 2007; 33: 667–701. doi: 10.1111/j.1728-4457.2007.00193.x

[3] UNFPA: UN Populations Fund. State of the World Population 2007; Unleashing the Potential of Urban Growth. New York, UNFPA 2007.

[4] Floro MS, Swain RB. Food Security, Gender, and Occupational Choice among Urban Low-Income Households. World Dev 2013; 42: 89–99. doi: 10.1016/j.worlddev.2012.08.005

[5] Maitra C, Rao DSP. Poverty-Food Security Nexus: Evidence from a Survey of Urban Slum Dwellers in Kolkata. World Dev. 2015; 72: 308–325. doi: 10.1016/j.worlddev.2015.03.006

[6] de Zeeuw H, van Veenhuizen R, Dubbeling M. The role of urban agriculture in building resilient cities in developing countries. J Agric Sci. 2011; 149: 153–163. http://dx.doi.org/10.1017/S0021859610001279

[7] Poulsen MN, McNab PR, Clayton ML, Neff RA. A systematic review of urban agriculture and food security impacts in low-income countries. Food Policy. 2015; 55: 131–146. doi: 10.1016/j.foodpol.2015.07.002

[8] Thys E, Oueadraogo M, Speybroeck N, Geerts S. Socio-economic determinants of urban household livestock keeping in semi-arid Western Africa. J Arid Environ. 2005; 63: 475–496. doi: 10.1016/j.jaridenv.2005.03.019

[9] Randolph TF, Schelling E, Grace D, Nicholson CF, Leroy JL, Cole DC, et al Invited review: Role of livestock in human nutrition and health for poverty reduction in developing countries. J Anim Sci. 2007; 85: 2788–2800. doi: 10.2527/jas.2007-0467 17911229 10.2527/jas.2007-0467

[10] Warren E, Hawkesworth S, Knai C. Investigating the association between urban agriculture and food security, dietary diversity, and nutritional status: A systematic literature review. Food Policy. 2015; 53: 54–66. doi: 10.1016/j.foodpol.2015.03.004

[11] Zezza A, Tasciotti L. Urban agriculture, poverty, and food security: Empirical evidence from a sample of developing countries. Food Policy. 2010; 35: 265–273. doi: 10.1016/j.foodpol.2010.04.007

[12] FAO. Urban and peri-urban agriculture: A briefing guide for the successful implementation of Urban and peri-urban agriculture in developing countries and countries of transition. Special Programme for Food Security. 2001.

[13] Makita K, Fevre EM, Waiswa C, Kaboyo W, Eisler MC, Welburn SC. Evidence-Based Identification of the Most Important Livestock Related Zoonotic Diseases in Kampala, Uganda. J Vet Med Sci. 2011; 73: 991–1000. 21467754 10.1292/jvms.11-0049

[14] Kang'ethe EK, Grace D, Randolph TF. Overview on urban and peri-urban agriculture: definition, impact on human health, constraints and policy issues. East Afr Med J. 2007; 84: 48–56. 18338722 10.4314/eamj.v84i11.9576

[15] Vu TKV, Tran MT, Dang TTS. A survey of manure management on pig farms in Northern Vietnam. Livest Sci. 2007; 112: 288–297. doi: 10.1016/j.livsci.2007.09.008

[16] Huong LQ, Madsen H, Anh le X, Ngoc PT, Dalsgaard A. Hygienic aspects of livestock manure management and biogas systems operated by small-scale pig farmers in Vietnam. Sci Total Environ. 2014; 470–471: 53–57. doi: 10.1016/j.scitotenv.2013.09.023 24140681 10.1016/j.scitotenv.2013.09.023

[17] FAO/OIE/World Bank. Good practices for biosecurity in the pig sector—Issues and options in developing and transition countries. FAO Animal Production and Health 2010. Paper No. 169.

[18] Robinson TP, Wint GRW, Conchedda G, Van Boeckel TP, Ercoli V, Palamara E, et al Mapping the Global Distribution of Livestock. PloS ONE. 2014; 9(5): e96084 doi: 10.1371/journal.pone.0096084 24875496 10.1371/journal.pone.0096084 PMC4038494

[19] Fualefac DH, Raphae KJ, Bime MJ, Ndebi G, Yemele F, Zoli PA, et al Socioeconomic and technical characteristics of pig farming in the urban and peri-urban zone of Dschang—West region of Cameroon. Discourse Journal of Agriculture and Food Science. 2014; 2: 11–20. Available: http://www.resjournals.org/JAFS/PDF/2014/Jan/Fualefac_et_al.pdf.

[20] Kambashi B, Picron P, Boudry C, Théwis A, Kiatoko H, Bindelle J. Smallholder pig production systems along a periurban-rural gradient in the Western provinces of the Democratic Republic of the Congo. Journal of Agriculture and Rural Development in the Tropics and Subtropics. 2014; 115: 9–22. Available: http://www.jarts.info/index.php/jarts/article/view/2014020344851.

[21] Adesehinwa AOK, Aribido SO, Oyediji GO, Obiniyi AA. Production strategies for coping with the demand and supply of pork in some peri-urban areas of Southwestern Nigeria. Livestock Research for Rural Development. 2003; 15: Article #72. Available: http://www.lrrd.org/lrrd15/10/ades1510.htm.

[22] Bank World. World Development Indicators 2014. http://databank.worldbank.org/data/reports.aspx?source=world-development-indicators.

[23] Faugier J, Sargeant M. Sampling hard to reach populations. J Adv Nurs. 1997; 26: 790–797. 9354993 10.1046/j.1365-2648.1997.00371.x

[24] FAO. The State of Food and Agriculture 2014. Innovation in family farming. 2014. http://www.fao.org/publications/sofa/2014/en/.

[25] Swedish Code of Statutes. The Act concerning the Ethical Review of Research Involving Humans (2003:460). https://www.riksdagen.se/sv/Dokument-Lagar/Lagar/Svenskforfattningssamling/Lag-2003460-om-etikprovning_sfs-2003-460/.

[26] Howe LD, Galobardes B, Matijasevich A, Gordon D, Johnston D, Onwujekwe O, et al Measuring socio-economic position for epidemiological studies in low- and middle-income countries: a methods of measurement in epidemiology paper. Int J Epidemiol. 2012; 41: 871–886. doi: 10.1093/ije/dys037 22438428 10.1093/ije/dys037 PMC3396323

[27] Filmer D, Pritchett LH.Estimating wealth effects without expenditure data—or tears: an application to educational enrollments in states of India. Demography. 2001; 38: 115–132. 11227840 10.1353/dem.2001.0003

[28] Vyas S, Kumaranayake L. Constructing socio-economic status indices: how to use principal components analysis. Health Policy Plan. 2006; 21: 459–468. doi: 10.1093/heapol/czl029 17030551 10.1093/heapol/czl029

[29] Howe LD, Hargreaves JR, Huttly SR. Issues in the construction of wealth indices for the measurement of socio-economic position in low-income countries. Emerg Themes Epidemiol. 2008; 5: 3 doi: 10.1186/1742-7622-5-3 18234082 10.1186/1742-7622-5-3 PMC2248177

[30] Andersson Djurfeldt A. Multi-Local Livelihoods and Food Security in Rural Africa. J Int Dev. 2015; 27: 528–545. doi: 10.1002/jid.2991

[31] Samkol P, Borin K, Sovann S. Pig systems in Southeast Asia—the case of Cambodia. In: Thorpe W, Jemaneh T, editors. In: Pig systems in Asia and the Pacific: how can research and development enhance benefits to the poor? Proceedings of a regional conference held 23–24 November at Bangkok, Thailand. Nairobi, Kenya: International Livestock Research Institute. 2008: pp. 34–42.

[32] Osbjer K, Boqvist S, Sokerya S, Kannarath C, San S, Davun H, et al Household practices related to disease transmission between animals and humans in rural Cambodia. BMC Public Health. 2015; 15: 476 doi: 10.1186/s12889-015-1811-5 25952633 10.1186/s12889-015-1811-5 PMC4427931

[33] Saroeun K, Sokerya S, Samkol P, Ty C, Theara S, Sunnara S, et al. Assessment of pig production, feed and feeding practices in three main agro-ecological zones of Cambodia. In: Preston R, Ogle B, editors. Proceedings of Regional Conference 2007: Matching livestock systems with available resources. Ha Long Bay, Vietnam. http://mekarn.org/training/courses/matching-livestock-systems-with-available-resources-3.

[34] Ajala MK, Adesehinwa AOK, Mohammed AK. Characteristics of smallholder pig production in southern Kaduna area of Kaduna State, Nigeria. American-Eurasian J Agric & Environ Sci. 2007; 2: 182–188.

[35] Phengsavanh P, Ogle B, Stur W, Frankow-Lindberg BE, Lindberg JE. Smallholder Pig Rearing Systems in Northern Lao PDR. Asian Australas J Ani Sci. 2011; 24: 867–874.

[36] Lemke U, Kaufmann B, Thuy LT, Emrich K, Zarate AV. Evaluation of smallholder pig production systems in North Vietnam: Pig production management and pig performances. Livest Sci. 2006; 105: 229–243. doi: 10.1016/j.livsci.2006.06.012

[37] Chittavong M, Lindberg JE, Jansson A. Feeding regime and management of local Lao pigs in Central Lao PDR. Trop Ani Health Prod. 2013; 45: 149–155. doi: 10.1007/s11250-012-0186-1 22639036 10.1007/s11250-012-0186-1

[38] Katongole CB, Sabiiti E, Bareeba F, Ledin I. Utilization of Market Crop Wastes as Animal Feed in Urban and Peri-Urban Livestock Production in Uganda. J Sustainable Agric. 2011; 35: 329–342. doi: 10.1080/10440046.2011.554318

[39] Assana E, Amadou F, Thys E, Lightowlers MW, Zoli AP, Dorny P, et al Pig-farming systems and porcine cysticercosis in the north of Cameroon. J Helminthol. 2010; 84: 441–446. doi: 10.1017/S0022149X10000167 20334716 10.1017/S0022149X10000167 PMC2965494

[40] Ouma E, Dione M, Lule P, Roesel K, Pezo D. Characterization of smallholder pig production systems in Uganda: constraints and opportunities for engaging with market systems. Livestock Research for Rural Development. 2014; 26: Article #56. Available: http://www.lrrd.org/lrrd26/3/ouma26056.htm

[41] Bonfoh B, Schwabennauer K, Wallinga D, Hartung J, Schelling E, Zinsstag J, et al Human health hazards associated with livestock production In: Steinfeld H, Mooney HA, Schneider F, Neville LE, editors. Livestock in a changing landscape. Washington: Island press; 2010 pp. 197–219.

[42] Huynh TTT, Aarnink AJA, Drucker A, Verstegen MWA. Pig production in Cambodia, Laos, Philippines and Vietnam: A review. Asian Journal of Agriculture and Development. 2007; 3: 69–90.

[43] Chan M. Food safety must accompany food and nutrition security. Lancet. 2014; 384: 1910–1911. doi: 10.1016/S0140-6736(14)62037-7 25467592 10.1016/S0140-6736(14)62037-7

[44] FAO. Livestock keeping in urban areas—A review of traditional technologies based on literature and field experience. FAO Animal Production and Health 2001. Paper No. 151.

