# Supplementary material for: Urban and peri-urban family-based pig-keeping in Cambodia: Characteristics, management and perceived benefits and constraints
Source: PLoS One. 2017 Aug 16;12(8):e0182247. doi: 10.1371/journal.pone.0182247 (PMC5559072; doi:10.1371/journal.pone.0182247)
Supplement: S1 Questionnaire — (DOCX) [file pone.0182247.s001.docx]

# Urban and peri-urban livestock-keeping in Cambodia

## Identification

| 1.1. Questionnaire ID |  | |
| --- | --- | --- |
| 1.2. Date of Survey (DD/MM/YYYY) |  | |
| 1.3. Khan  1.4 Commune  1.5 Village |  | |
| 1.6 GPS Coordinates | Latitude (N/S): | Longitude (E/W): |

## Household members

2.1. Details of household members (including the household head). [*WE DEFINE A “HOUSEHOLD” TO INCLUDE ALL MEMBERS OF A COMMON DECISION MAKING UNIT (USUALLY WITHIN ONE RESIDENCE) THAT ARE SHARING INCOME AND OTHER RESOURCES]*

|  | **Members of the household** | **Age** | **Gender**  (1=Male 2=Female) | **Highest education level attained** | **School on-going**  (Tick if yes) | **Literacy**  (1=Yes 2=No) | **Main occupation** |
| --- | --- | --- | --- | --- | --- | --- | --- |
| 1 |  |  |  |  |  |  |  |
| 2 |  |  |  |  |  |  |  |
| 3 |  |  |  |  |  |  |  |
| 4 |  |  |  |  |  |  |  |
| 5 |  |  |  |  |  |  |  |
| 6 |  |  |  |  |  |  |  |
| 7 |  |  |  |  |  |  |  |
| 8 |  |  |  |  |  |  |  |
| 9 |  |  |  |  |  |  |  |
| 10 |  |  |  |  |  |  |  |
| **Household members**  1 = Head  2 = Spouse  3 = Son  4 = Daughter  5 = Sibling  6 = Parent  7 = Grandchild  8 = Other relative  9 = Non-relative (including employees who live in house)  10 = Other, specify__________________ | | | **Highest education level attained**  0 = No formal education  1 = Nursery- Pre-school age  2 = Primary education  3 = Lower Secondary school (7-9^th^ grade)  4 = Upper Secondary school (10-12^th^ grade)  5 = Vocational training (specify no of yrs.)_________  6 = Tertiary training specify yrs. _________  7 = University degree (undergraduate)  8 = University degree (postgraduate)  9=Other, specify ________________________ | | | **Main occupation**  0 = None  1 = Crop farming  2 = Pig keeping  3 = Cattle keeping  4 = Poultrykeeping  5 = Mixed farming (crop+animal)  6 = Self-employed-off farm  7 = Other, specify_______________  8 = Rice vine production | |
| ***Indicate who the respondent (R) is and who is responsible for taking care of the livestock (LK).*** | | | | | | | |

## Household information

| 3.1 | Does the household own the house? | *[1] =Yes [ 2]=No (residential building)* |
| --- | --- | --- |
| 3.2 | Does the household have access to any agricultural land which is used for growing crops, keeping livestock etc.? | *[1] =Yes [ 2]=No* |
| 3.3 | If yes, how large is the area of this land?  Re-calculation: ___________________ ha | *Recalculate into hectares after the survey* |
| 3.4 | Does the household own that land, or parts of that land? | *[1] =Own all land*  *[2]=Rent all land*  *[3]=Own part of that land*  *[4]= Other, specify* |
| 3.5 | What kind of production is the land used for? |  |
| 3.6 | What are the main sources of income for the household? | *____________________________* |
| 3.7 | Wall type of the house | *[1]=Brick/Block/Concrete*  *[2]=Wood and Concrete*  *[3]=Wood*  *[4]=Wattle/Tapia/Makeshift*  *[5]= Poor wood/zinc*  *[6]= Other, specify* |
| 3.8 | Type of floor | *[1]=Concrete*  *[2]=Earth*  *[3]=Stone tiles*  *[4]=Wooden*  *[5]=Other, specify* |
| 3.9 | Type of roof | *[1]=Zinc*  *[2]=Wood*  *[3]=Tiled*  *[4]=Leaf*  *[5]=Other, specify* |
| 3.10 | Area of the house (residential building)? | *Write the area in m^2^ and indicate if* |
| 3.11 | Number of rooms in the household |  |
| 3.12 | How many persons sleep in each bedroom? | *[1]= 1(incl. couple)*  *[2]= 2-3*  *[3]= 4 or more* |
| 3.13 | Sanitation facilities | *[1]=Private flush toilet*  *[2]=Public flush toilet*  *[3]=Pit latrine*  *[4]= Go to toilet in other’s house*  *[5]= Go to field*  *[6]= Other, specify* |
| 3.14 | Do you have access to electricity? | *[1]=Yes [2]=No* |
| 3.15 | What do you use as cooking fuel? | *[1]=Electricity*  *[2]=Wood*  *[3]=Dung*  *[4]=Charcoal*  *[5]=Kerosene*  *[6]=Gas*  *[7]=Biogas*  *[8]=Other, specify* |
| 3.16 | What kind of vehicle(s) do you have in the household?   - Car - Pickup truck - Tuktuk - Motorbike - Bicycle - Hand-tractor - Cart - Other, specify | *Write number of each type of vehicle* |
| 3.17 | Please indicate the household appliances that you have in the household.   - Stove - Refrigerator - Washing machine - TV - Radio - Computer - Mobile phone - Sewing machine - Air condition | *If present, write the amount* |
| 3.18.  3.19 | What is your main source of drinking water for human consumption in the…   - Dry season - Rainy season | *[1]=Bottled water*  *[2]=Tap water in residence*  *[3]=Tap water in public tap*  *[4]=Well in residence*  *[5]=Public well*  *[6]=River, canal or surface water*  *[7]=Rainwater*  *[8]=Water from truck*  *[9]=Other source, specify* |
| 3.20 | How do you treat the drinking water for human consumption?*(unless they only drink bottled water)* | *[1]=Boiled*  *[2]=Filtered*  *[3]= Boiled and filtered*  *[4]= Not treated*  *[5]= Other, specify* |
| 3.21  3.22 | What is your main source of drinking water for animal consumption in the…   - Dry season - Rainy season | *[1]=Tap water in residence*  *[2]=Tap water in public tap*  *[3]=Well in residence*  *[4]=Public well*  *[5]=Spring for drinking water*  *[6]=River, canal or surface water*  *[7]=Rainwater*  *[8]=Water from truck*  *[9]=Other source, specify* |

## Livestock in the household

| 4.1 | Does the household own the livestock in the household? | *[1]=Yes,*  *[2]=No*  *[3]=Own some, lend some*  *[4]=Lend all animals*  *[5]= Other, specify* |
| --- | --- | --- |
| 4.2 | Who in the household owns the livestock? | *[1]=Household head*  *[2]=Spouse*  *[3]=Both/all*  *[4]=Other male hh member*  *[5]=Other female hh member*  *[6]=Other, specify* |
| 4.3 | For how long have you been keeping animals? |  |
| 4.4 | Did you or any other member in your household have any previous experience in animal keeping? | *[1]=Yes [2]=No* |
| 4.5 | If yes, what kind of experience? |  |

4.6 Indicate the numbers of animals for the different species kept in the household and how they are being housed. Several alternatives are possible.

| **Livestock** | **Number kept by the household** | **Animals kept in pen** | **Animals tied up** | **Combination of free roaming and confined** | **Free roaming** |
| --- | --- | --- | --- | --- | --- |
| Cattle |  |  |  |  |  |
| Pig |  |  |  |  |  |
| Chicken |  |  |  |  |  |
| Ducks |  |  |  |  |  |
| Buffaloes |  |  |  |  |  |
| Donkeys/Horses |  |  |  |  |  |
| Dogs |  |  |  |  |  |
| Cats |  |  |  |  |  |
| Other, specify |  |  |  |  |  |

4.7 Indicate the different categories of cattle kept currently

| **Type** | **Breed type** | **Numbers kept** |
| --- | --- | --- |
| Cows |  |  |
| Bulls (> 1 year) |  |  |
| Heifers (>1 year) |  |  |
| Calves (Bulls and heifers <1 year) |  |  |
|  | **Breed type:**  1 = Local; 2 = Exotic; 3 = Cross | |

4.8 Indicate the different categories of pigs kept currently

| **Type** | **Breed type** | **Numbers kept** |
| --- | --- | --- |
| Breeding boars |  |  |
| Breeding sows |  |  |
| Fatteners (>3 months) |  |  |
| Growers (1-3 months) |  |  |
| Piglets (<1 month) |  |  |
|  | **Breed type:**  1 = Local; 2 = Exotic; 3 = Cross | |

4.9 Have any livestock left your herd (or died) in the last 12 months?  *[1]=Yes, [2]=No*
4.10 If yes, fill in the table below:

| **Type**  (code) | **How exited** (code) | **How many animals exited?** | **If sold, where?**  (code) | **Purpose of selling?**(code) |
| --- | --- | --- | --- | --- |
|  |  |  |  |  |
|  |  |  |  |  |
|  |  |  |  |  |
|  |  |  |  |  |
|  |  |  |  |  |
|  |  |  |  |  |
|  |  |  |  |  |
|  |  |  |  |  |
|  |  |  |  |  |
|  |  |  |  |  |
| **Type**  1 = Breeding boars  2 = Breeding sows  3 = Fatteners  4 = Growers  5 = Piglets  6 = Cows  7 = Bulls  8 = Calves (<1yr)  9 = Heifers | **How exited**  1 = Sold  2 = Death  3= Gift  4= Other, specify | | **Where sold**  1 = At household to trader/middleman/ slaughter house  2 = Market  3 = At slaughter house  4 = Other, specify | **Purpose of selling**  1 = To meet planned household expenses  2 = To meet emergence household expenses  3 = Livestock trading as a business  4= Animal was sick  5 = Other, specify |

4.11 Has there been any inflow of livestock (pigs or cattle) through purchases, births or any other form in the last 12 months? *[1]=Yes, [2]=No*

4.12 If yes, please provide the details:

| **Type**  (code) | **Breed**  (code) | **Type of entry**  (code) | **No. of animals** |
| --- | --- | --- | --- |
|  |  |  |  |
|  |  |  |  |
|  |  |  |  |
|  |  |  |  |
|  |  |  |  |
|  |  |  |  |
|  |  |  |  |
|  |  |  |  |
| **Type**  1 = Breeding boars  2 = Breeding sows  3=Fatteners  4 = Growers  5 = Piglets  6=Cows  7=Bulls  8=Calves (<1yr)  9=Heifers | **Breed**  1= Local  2= Exotic  3= Cross | **Type of entry**  1 = Bought  2 = Loan or gift from project  3= Gift from others  4= Birth/ born on farm  5=Other, specify_________________________ | |

## Manure management

| 5.1  5.2 | Do you collect the manure from the…   - Cattle? - Pigs? | *[1]=Yes [2]=No* |  |
| --- | --- | --- | --- |
| 5.3  5.4  5.5  5.6  5.7  5.8 | What do you do with the cattle manure?  Store it, use for  crops/veg’s  Store it, sell/give away later  Store it, not use  Take to fish pond __________  Discharge it  Other, specify | *[1]=Yes [2]=No* | *If cattle.* |
| 5.9  5.10  5.11  5.12  5.13  5.14 | What do you do with the pig manure?  Store it, use for  crops/veg’s  Store it, sell/give away later  Store it, not use  Take to fish pond __________  Discharge it  Other, specify | *[1]=Yes [2]=No* |  |
| 5.15  5.16 | How do you clean the pig pens?  Scrape solid  Hose with water | *[1]=Yes [2]=No* | *If pigs* |
| 5.17 | How often do you clean the pig pens? | *[1]=Several times a day*  *[2]=Once a day*  *[3]=Every second day*  *[4]=Less than twice a week* | *If pigs* |
| 5.18  5.19 | Do you use any disinfectants?   - Cattle - Pig | *[1]=Yes [2]=No* |  |
| 5.20  5.21 | If yes, how often?   - Cattle - Pig | *[1]=Several times a week*  *[2]=Once a week*  *[3]=Every second week*  *[4]=Once a month*  *[5]=Every 3 months*  *[6]=Every 6 months (cycle)*  *[7]=Other, specify* |  |
| 5.22  5.23  5.24  5.25  5.26 | If you store the manure, how is it stored?  In a lagoon/pond  In a pile with  fences  In a pile without  fences  In bags  Other, specify | *[1]=Yes [2]=No* | *Only if they store the manure in some way* |
| 5.27  5.28 | For how long is the manure being stored during…  -Dry season  -Rainy season | *[1]=More than 3 months*  *[2]=1-3 months*  *[3]=Less than 1 month*  *[4]=Used/discharged im.*  *[5]= Stored for about 1 yr*  *[6]= Never emptied*  *[7]= Other, specify* | *Only if they collect the manure in some way* |
| 5.29 | Do you treat the manure in some way? | *[1]=Yes [2]=No* |  |
| 5.30 | If yes, how do you treat the manure? |  |  |
| 5.31  5.32  5.33  5.34 | If you do not use the manure, how do you discharge it?  Into environment  Into lake  Into river  Other, specify | *[1]=Yes [2]=No* | *Should also be answered by farmers that use a bio digester. The effluent might be discharged* |
| 5.35 | Why don’t you use the  manure? |  | *Only if they do not use the manure* |
| 5.36 | Distance of manure storage to human drinking water? | *Write the distance in meters.* | *Only if other water sources than tap and bottled water* |
| 5.37 | Do you protect yourself when being in contact with animals or manure? | *[1]=Yes [2]=No* |  |
| 5.38  5.39  5.40  5.41  5.42 | If yes, what precautions do you take?  Wash hands with water and soap: ______________  Use mask ______________  Use protective  clothing ______________  Use gloves ______________  Use boots ______________  Other, specify ______________ | *[1]=Yes [2]=No* |  |

1. **Feed**

| 6.1  6.2  6.3  6.4  6.5  6.6  6.7  6.8  6.9  6.10 | What kinds of feed do you provide the cattle?  Pasture  Forage  Rice bran  Broken rice  Rice full grain  Rice straw, own  Rice straw from market ________  Banana stem, own ________  Banana stem  from market _________  Other, specify | *[1]=Yes [2]=No* | *If cattle* |
| --- | --- | --- | --- |
| 6.11  6.12  6.13  6.14  6.15  6.16  6.17  6.18  6.19  6.20  6.21  6.22  6.23  6.24 | What kinds of feed do you provide the pigs?  Rice bran  Broken rice  Rice full grain  Rice vine residues  Dried rice  Kitchen waste  Water spinach, own ________  Water spinach fr market  Waste from restaurants  and food shops  Concentrate  Banana stem, own __________  Banana stem fr market _________  Brewery residues ________  Other, specify | *[1]=Yes [2]=No* | *(other than dried rice)* |
| 6.25 | Do you use any additives to the feed? Ex antibiotics, growth promoters etc. | *[0]=I don’t know*  *[1]=Yes*  *[2]=No* |  |
| 6.26 | If yes, what kinds of additives? | *[0]=I don’t know* |  |
| 6.27 | Do you ever feed the pigs uncooked meat waste? | *[1]=Yes [2]=No* |  |

1. **Animal health**

| 7.1  7.2 | Have you had any diseases among the pigs/cattle in the last three years?   - Cattle - Pigs | *[1]=Yes [2]=No* |
| --- | --- | --- |
| 7.3  7.4 | If yes, what kind of diseases?   - Cattle - Pigs | *[0]=I don’t know* |
| 7.5  7.6 | If don’t know [0], what were the symptoms?  -Cattle  -Pigs |  |
| 7.7  7.8 | Do you vaccinate the animals?   - Cattle - Pigs | *[1]=Yes [2]=No* |
| 7.9  7.10  7.11  7.12  7.13  7.14  7.15  7.16  7.17  7.18  7.19  7.20 | If yes, against what diseases?  Cattle  FMD  Blackleg  Pasteurellosis (HS)  Other, specify  Pig  FMD  PRRS  Pestis suum (Classical Swine fever)  Pasteurellosis (HS)  Edema disease  Aujeszky’s disease  (Pseudorabies virus- PRV)  White diarrhea  Other, specify | *[0]=I don’t know*  *[1]=Yes*  *[2]=No*  *[0]=I don’t know*  *[1]=Yes*  *[2]=No* |
| 7.21  7.22 | Are the animals dewormed?   - Cattle - Pigs | *[1]=All animals*  *[2]=No animals*  *[3]=Some animals* |
| 7.23 | Do you think diseases can be transmitted from animals to humans? | *[1]=Yes [2]=No* |
| 7.24 7.25 7.26 | What do you do if an animal gets sick?   - Cattle - Pig - Chicken | *[1]=Treat it first, then call vet*  *[2]=Treat it themselves*  *[3]=The animal is not treated*  *[4]= Always call vet*  *[5]= Never sick*  *[6]= Other, specify* |
| 7.27  7.28  7.29 | What do you do with the remains of *most* sick animals? (animals that have not died on their own)   - Cattle - Pig - Chicken | *[1]=Most animals are killed and eaten*  *[2]=Kill and burned or buried*  *[3]=Kill and sold/given away*  *[4]= Kill and small ones are burned/buried, big ones are eaten*  *[5]= Kill and small ones are burned/buried, big ones are sold/given*  *[6]=Most sick animals are sold live*  *[7]=The animal is kept until it dies*  *[8]= Never sick*  *[9]= Always recover*  *[10]= Other, specify* |
| 7.30  7.31  7.32 | What do you do with fallen animals (animals that have died on their own)?   - Cattle - Pig - Chicken | *[1]=Most are burned/buried*  *[2]=Small animals are burned/buried, big ones are eaten*  *[3]=Most animals are eaten*  *[4]=Most are sold/given away*  *[5]=Small ones are sold/given away, big ones are eaten*  *[6]= Big ones are sold/given away, small ones are burned/buried*  *[7]= Never died*  *[8]= Other, specify* |

## Public health

| 8.1 | Do you or any other household member ever consume any undercooked meat? | *[1]=Yes [2]=No* |
| --- | --- | --- |
| 8.2 | If no, why not? |  |
| 8.3 | Do you or any other household member ever drink raw milk? | *[1]=Yes [2]=No* |
| 8.4 | If no, why not? |  |
| 8.5 | Have any household member had any gastrointestinal symptoms during the last 2 weeks? | *[1]=Yes [2]=No* |
| 8.6  8.7  8.8  8.9  8.10 | If yes, what kinds of symptoms?  Vomiting  Diarrhea  Nausea  Abdominal pain  Other, specify | *[1]=Yes [2]=No* |
| 8.11 | Do you know what caused the symptoms? | *[1]=Yes [2]=No* |
| 8.12 | If yes, what? |  |

## Impacts and livelihoods

9.1 What is/are the main reason(s) for your household to keep livestock?

9.2 In what way does keeping livestock affect your livelihoods?

9.3 What main factors may have a negative effect on your livestock production?
