# Supplementary material for: Urban and peri-urban family-based pig-keeping in Cambodia: Characteristics, management and perceived benefits and constraints
Source: PLoS One. 2017 Aug 16;12(8):e0182247. doi: 10.1371/journal.pone.0182247 (PMC5559072; doi:10.1371/journal.pone.0182247)
Supplement: S1 Table — (DOCX) [file pone.0182247.s002.docx]

# Self-reported household belongings and score of the principal component from the PCA used for the socio-economic index calculations

| **Household belonging** | **Principal Component score** |
| --- | --- |
| Household owns the residential building | 0.258 |
| Access to agricultural land | 0.357 |
| Wall type of the house  Brick/block/concrete  Wood and concrete  Wood  Zinc/broken wood  Wattle | 0.258  0.547  -0.304  -0.424  -0.389 |
| Floor type of the house  Stone tile  Wood  Concrete  Earth | 0.635  -0.184  -0.179  -0.503 |
| Roof type of the house  Tiles  Zinc  Wood  Leaf | 0.662  -0.628  0.044  -0.182 |
| Three persons or less per bedroom | 0.380 |
| Flush toilet in residence | 0.599 |
| Access to electricity | 0.218 |
| Type of cooking fuel  Electricity  Gas  Biogas  Charcoal  Kerosene  Wood | 0.021  0.190  0.157  -0.107  0.044  -0.211 |
| Vehicles owned by the household  Car or pickup  Tuktuk  Motorbike  Bicycle  Hand-tractor  Cart | 0.232  0.073  0.197  0.172  0.099  0.315 |
| Assets owned by the household  Stove  Refrigerator  Washing machine  TV  Radio  Computer  Mobile phone  Sewing machine  Air condition | 0.295  0.382  0.343  0.311  0.253  0.288  0.186  0.053  0.297 |
| Main source of drinking water  Bottled water  Tap in residence  Tap in neighbour’s house  Well in residence  Public well  Truck water  Rain water  River, lake or surface water | -0.070  0.073  -0.153  -0.015  -0.045  -0.037  0.193  -0.041 |
| Ownership of cattle | 0.067 |
